# A broadly neutralizing germline-like human monoclonal antibody against dengue virus envelope domain III

**DOI:** 10.1371/journal.ppat.1007836

**Published:** 2019-06-26

**Authors:** Dan Hu, Zhongyu Zhu, Shun Li, Yongqiang Deng, Yanling Wu, Nana Zhang, Vinita Puri, Chunyu Wang, Peng Zou, Cheng Lei, Xiaolong Tian, Yulu Wang, Qi Zhao, Wei Li, Ponraj Prabakaran, Yang Feng, Jane Cardosa, Chengfeng Qin, Xiaohui Zhou, Dimiter S. Dimitrov, Tianlei Ying

**Affiliations:** 1 MOE/NHC/CAMS Key Laboratory of Medical Molecular Virology, School of Basic Medical Sciences, Shanghai Medical College, Fudan University, Shanghai, China; 2 National Cancer Institute, National Institutes of Health, Frederick, Maryland, United States of America; 3 Shanghai Public Health Clinical Center, Key Laboratory of Medical Molecular Virology of Ministries of Education and Health, Fudan University, Shanghai, China; 4 State Key Laboratory of Pathogen and Biosecurity, Beijing Institute of Microbiology and Epidemiology, Beijing, China; 5 Institute of Health and Community Medicine, Universiti Malaysia Sarawak, Malaysia; Icahn School of Medicine at Mount Sinai, UNITED STATES

## Abstract

Dengue is the most widespread vector-borne viral disease caused by dengue virus (DENV) for which there are no safe, effective drugs approved for clinical use. Here, by using sequential antigen panning of a yeast antibody library derived from healthy donors against the DENV envelop protein domain III (DIII) combined with depletion by an entry defective DIII mutant, we identified a cross-reactive human monoclonal antibody (mAb), m366.6, which bound with high affinity to DENV DIII from all four DENV serotypes. Immunogenetic analysis indicated that m366.6 is a germline-like mAb with very few somatic mutations from the closest VH and Vλ germline genes. Importantly, we demonstrated that it potently neutralized DENV both *in vitro* and in the mouse models of DENV infection without detectable antibody-dependent enhancement (ADE) effect. The epitope of m366.6 was mapped to the highly conserved regions on DIII, which may guide the design of effective dengue vaccine immunogens. Furthermore, as the first germline-like mAb derived from a naïve antibody library that could neutralize all four DENV serotypes, the m366.6 can be a tool for exploring mechanisms of DENV infection, and is a promising therapeutic candidate.

## Author summary

Dengue virus infects 50–100 million people each year. Infection is initiated by entry of the virus into cells mediated by the viral envelope glycoproteins. There are four closely related DENV serotypes, but they all are antigenically distinct, with each comprising several genotypes that exhibit differences in their infection characteristics in both the mosquito vector and in the human host. One of the confounding problems that has faced vaccine and biological drugs development for decades is the inability of antibodies to one serotype to protect against infection by another one. Instead, the induced humoral immune response to one dengue virus infection can enhance the infection and disease processes brought by a subsequent infection with another dengue serotype. In this study, by using a competitive sorting strategy to interrogate a human naïve antibody library, we identified a cross-reactive mAb, designated as m366.6, against the four DENV serotypes. The mAb m366.6 possesses only few somatic mutations from the closest VH and Vλ germline genes and high affinity to DIII. Most importantly, the germline-like m366.6 demonstrated a broad spectrum of neutralization against the four DENV serotypes. Thus, m366.6 is a promising candidate therapeutics and its epitope may imply on the design of effective vaccine immunogens to elicit m366.6-like antibodies *in vivo*.

## Introduction

Dengue virus (DENV) causes the most prevalent mosquito-borne viral disease. Over 2.5 billion people are at risk for infection in over 100 countries, 50–100 million are infected with symptoms, and up to 50,000 die from dengue hemorrhagic fever (DHF) and dengue shock syndrome (DSS) each year [1,2]. No specific antiviral drug has been available against DENV infection; the only approved vaccine, Dengvaxia, has caused considerable controversy regarding its safety and potential benefits [3–6]. For decades, anti-DENV vaccine and biological drugs development has been hampered by the high sequence divergence (25–40%) among the four DENV serotypes [7,8]. Such divergence leads to the fact that one antibody may not be sufficient to neutralize all DENV infection. Instead, the induced humoral immune response to one DENV infection can enhance the infection and disease processes brought by a subsequent infection with another DENV serotype [2–4]. These findings suggest that the development of new and broadly neutralization antibodies against all the serotypes of DENV could be promising candidate anti-DENV agents, and may also guide the design of effective and safe vaccine immunogens.

The DENV envelope glycoprotein (E protein), which mediates virus entry into cells, is the major neutralizing target of antibodies [9–13]. E protein is a type II fusion protein and consists of three domains: DI, DII, and DIII of which DIII has been proposed to contain a receptor binding domain [14–17]. Recent studies revealed that cross-reactive conserved epitopes exist on DII as well as DIII of the DENV E protein [14,16–18]. During the naturally-occurring primary DENV infection, a large fraction of the antibody repertoire consists of DII-specific antibodies which are, unfortunately, typically poor in neutralization and may increase the likelihood of severe disease upon subsequent infection through a mechanism known as antibody-dependent enhancement (ADE) [18–20]. In contrast, antibodies targeting DIII have proven to be the most potent neutralizing antibodies, but very few could be elicited in naturally infected individuals [18,19,21–35]. Despite this, previous studies indicated that anti-DENV DIII serotype-specific and cross-reactive antibodies could be elicited using DENV DIII as vaccine immunogen [36–43] and in infected humans [44–47]. It has also been demonstrated that the lysine at position 310 on DIII is the critical residue in the cross-reactive epitope [24]. Therefore, the conserved epitope on DIII represents an attractive target for the development of broadly neutralizing DENV antibodies.

Here, we report the isolation of a potent DENV DIII-specific human monoclonal antibody (mAb), designated as m366.6, from a large naïve antibody library constructed by the blood of healthy adult donors. A competitive sorting strategy using a DIII mutant as competitor was applied to identify antibodies precisely targeting the conserved neutralizing epitope. To our knowledge, m366.6 is the first human mAb isolated from a naïve antibody library which could neutralize all the four serotypes DENV viruses. Importantly, both heavy and light chain genes of m366.6 are very close to their putative germline predecessors. Its fully human origin, the germline-like nature, combined with high-affinity and broad neutralizing activity toward all DENV serotypes, suggest that m366.6 is a promising candidate antiviral agent and may also provide a unique template for designing effective dengue vaccine immunogens.

## Results

### Isolation and generation of DENV1-4 broadly neutralizing mAbs

**Fig 1:**
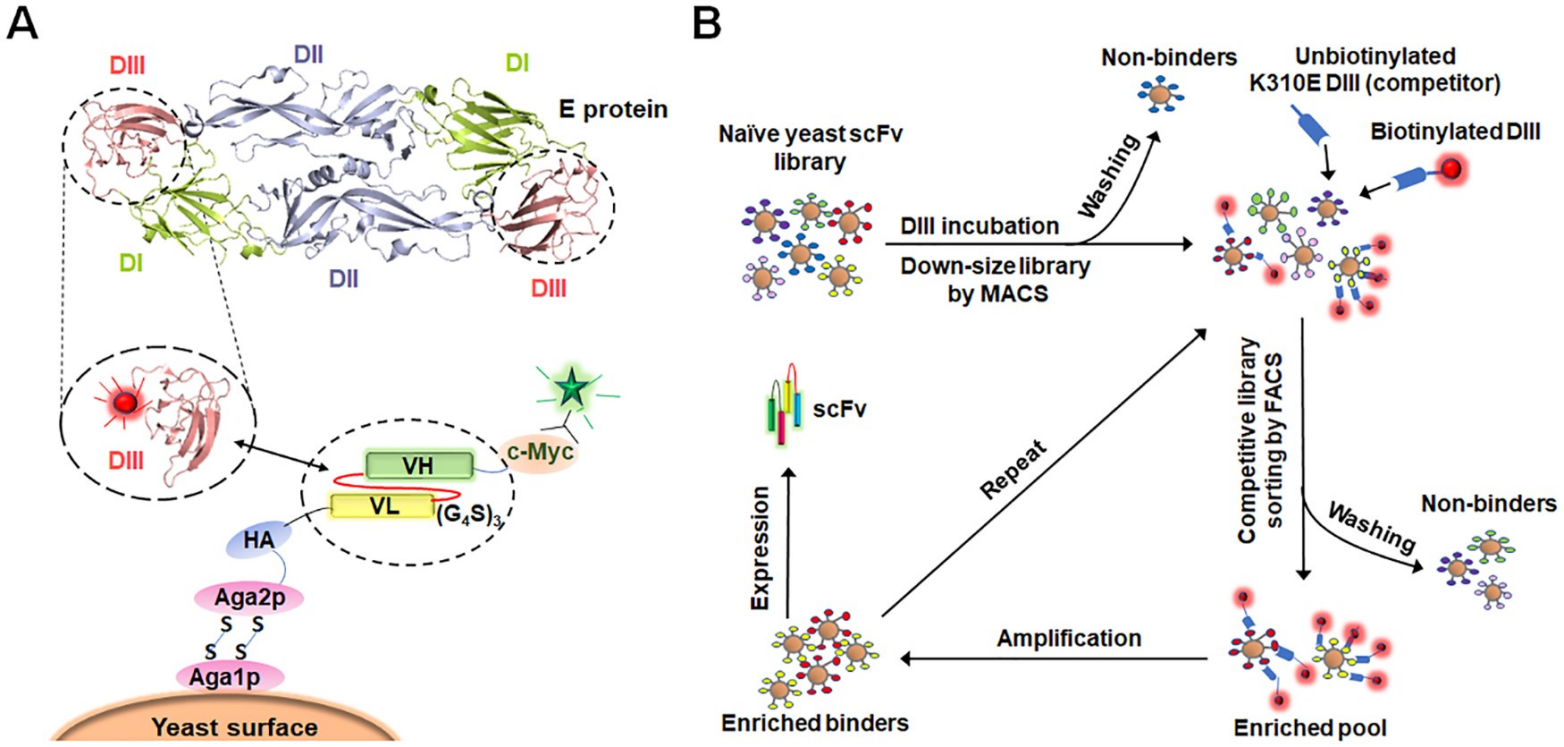
Yeast display-based bio-panning for the isolation of DENV DIII-specific antibodies. (A) The DENV E protein is consist of DI, DII and DIII, which are shown in green, cyan, and light pink, respectively. The scFv was displayed as an Aga2p (rose red) fusion protein on the surface of yeast. Antibody expression can be detected by using fluorescent anti-c-Myc antibody (green), and binding of the scFv to the biotinylated DIII can be detected using PE-conjugated streptavidin (red). HA, hemagglutinin; VL, variable light chain; VH, variable heavy chain; (G_4_S)_3_, (Gly_4_Ser)_3_-flexible peptide linker. (B) Schematic of the scFv panning process. The biotinylated DIII, shown with a fluorescent tag colored in red, was used as the antigen for bio-panning, and ten times concentration of unbiotinylated DIII K310E mutant was used as the competitor. MACS and FACS-based sorting strategies were used to isolate high affinity binders from the yeast-displayed antibody library.

We previously prepared some large naïve antibody libraries using peripheral blood B lymphocytes of non-immunized healthy donors and used them for panning/screening against viral and cancer targets [48–55]. In this study, we used a competitive library sorting strategy to isolate broadly neutralizing antibodies against DENV1-4 (Fig 1A and 1B). The yeast-displayed naïve single chain antibody fragment (scFv) library was used to screen against the biotinylated DENV DIII, and, importantly, ten times concentration unbiotinylated DIII K310E mutant was used as the competitor. The yeast cells were selected to present the antibody-expressing cells that could bind well to the wild-type DIII instead of the DIII mutant, resulting in the isolation of antibodies that can target the cross-reactive neutralizing epitopes covering the residue Lysine310 [55]. Potent enrichment was achieved after four rounds of sorting, and a panel of antibodies were identified (Fig 1B). Two antibodies, designated as m360 and m366, bound potently to DENV DIIIs. Their scFv gene were fused with human IgG1 Fc for protein expression, and surface plasmon resonance (SPR) experiments were used to evaluate the antigens binding. The equilibrium dissociation constant (K_D_) of m360 for the DENV1-4 DIIIs were 5.8 nM, 5.1 nM, 0.1 nM and 8.3 nM, respectively. The mAb m366 displayed a broader binding profile compared with that of m360, with the K_D_ of 3.3 nM, 1.2 nM, 1.1 nM and 12 nM to DENV1-4, respectively (Table 1, S1 and S2 Figs).

**Table 1:** Binding kinetics of DENV1-4 DIII-specific mAbs measured by Biacore.

		DENV-1 DIII	DENV-2 DIII	DENV-3 DIII	DENV-4 DIII
**m360**	***k***_**on**_ **(Ms**^**-1**^**)**	**1.2×10**^**6**^	**1.7×10**^**5**^	**5.9×10**^**5**^	**9.0×10**^**4**^
	***k***_**off**_ **(s**^**-1**^**)**	**7.0×10**^**−3**^	**8.8×10**^**−4**^	**6.9×10**^**−5**^	**7.5×10**^**−4**^
	**K**_**D**_ **(M)**	**5.8×10**^**−9**^	**5.1×10**^**−9**^	**1.2×10**^**−10**^	**8.3×10**^**−9**^
**m366**	***k***_**on**_ **(Ms**^**-1**^**)**	**7.6×10**^**5**^	**7.7×10**^**5**^	**1.9×10**^**5**^	**2.7×10**^**5**^
	***k***_**off**_ **(s**^**-1**^**)**	**2.5×10**^**−3**^	**9.3×10**^**−4**^	**2.0×10**^**−4**^	**1.0×10**^**−2**^
	**K**_**D**_ **(M)**	**3.3×10**^**−9**^	**1.2×10**^**−9**^	**1.1×10**^**−9**^	**1.2×10**^**−8**^
**m360.6**	***k***_**on**_ **(Ms**^**-1**^**)**	**1.1×10**^**6**^	**3.8×10**^**5**^	**4.1×10**^**5**^	**1.5×10**^**6**^
	***k***_**off**_ **(s**^**-1**^**)**	**3.4×10**^**−4**^	**1.6×10**^**−5**^	**9.4×10**^**−7**^	**5.3×10**^**−2**^
	**K**_**D**_ **(M)**	**3.1×10**^**−10**^	**4.2×10**^**−11**^	**2.3×10**^**−12**^	**3.3×10**^**−8**^
**m366.6**	***k***_**on**_ **(Ms**^**-1**^**)**	**3.2×10**^**5**^	**9.6×10**^**5**^	**6.6×10**^**5**^	**3.3×10**^**5**^
	***k***_**off**_ **(s**^**-1**^**)**	**2.5×10**^**−4**^	**2.5×10**^**−4**^	**1.8×10**^**−4**^	**6.2×10**^**−4**^
	**K**_**D**_ **(M)**	**7.8×10**^**−10**^	**2.9×10**^**−10**^	**2.7×10**^**−10**^	**1.9×10**^**−9**^

**Fig 2:**
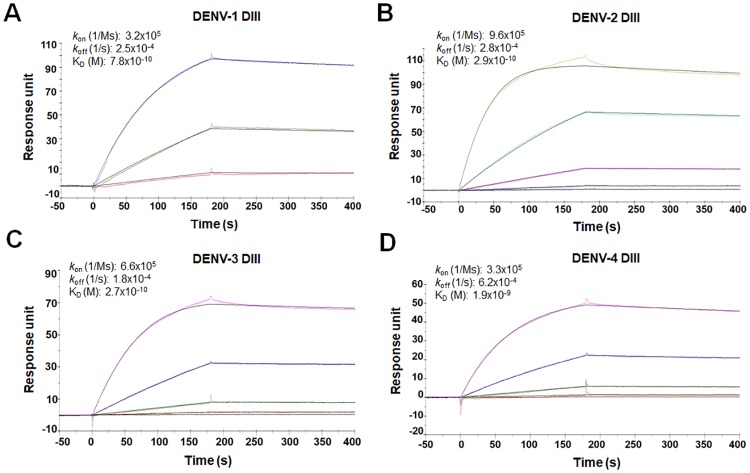
Binding of m366.6 to DENV DIIIs from the four serotypes measured by Biacore. The m366.6 was immobilized onto a CM5 chip, and the analytes consisted of serial dilution of DIII from DENV-1 (A), DENV-2 (B), DENV-3 (C), or DENV-4 (D). Binding kinetics was fitted using a 1:1 Langumir binding model by BIAevaluation 3.2 software.

To further improve the affinity of m360 and m366 with the four DENV serotypes, we constructed a mutant library using the error-prone PCR technologies. Following three cycles of mutagenesis and selection, two clones were identified from the enriched pool of yeast sorting, designated as m360.6 and m366.6. Biacore analysis showed that the cross-reactive binding activities of m360.6 and m366.6 to all 4 DIIIs were preserved after the affinity maturation process. The K_D_ of m360.6 for the DENV1-4 DIIIs were 0.3 nM, 42 pM, 2.3 pM and 33 nM, respectively (S3 Fig). Although the binding to DENV1-3 DIIIs was improved, the m360.6 had only slightly increased binding affinity to DENV4 DIII compared to its parental mAb m360. Notably, the m366.6 exhibited high affinity to all the DENV DIIIs. The K_D_ of m366.6 for the DENV1-4 were 0.8 nM, 0.3 nM, 0.3 nM, and 1.9 nM respectively, which demonstrated that m366.6 could bind to all the four serotype DENV viruses with high avidity (Table 1, Fig 2). We also assessed the binding specificity of m366.6 by ELISA, and the results showed that m366.6 had weak cross-reactivity with Zika virus (ZIKV) DIII and no binding with other irrelevant antigens (S4 Fig).

### Neutralization activity *in vitro*

Next, we assessed the neutralization capacity of m360.6 and m366.6 against the four DENV serotypes using a DENV luciferase reporter viral particle (RVP) neutralization assay. We used DENV RVPs against the four dengue serotypes that are common strains in DENV research: DENV-1 (WestPac 74), DENV-2 (S16803), DENV-3 (CH53489), and DENV-4 (TVP360). The luminescent reporter expression was proportional to the number of RVPs added to BHK DC-SIGN cells, confirming the linear correlation between the extent of RVP infection and reporter gene expression. In consistent with the Biacore binding results, both m360.6 and m366.6 could neutralize all the four serotype DENV, and m366.6 displayed better neutralization than m360.6, with the 50% neutralization titers (IC_50_) of 22, 2.4, 0.85, and 0.36 μg/ml against DENV1-4 respectively (S5 Fig).

**Fig 3:**
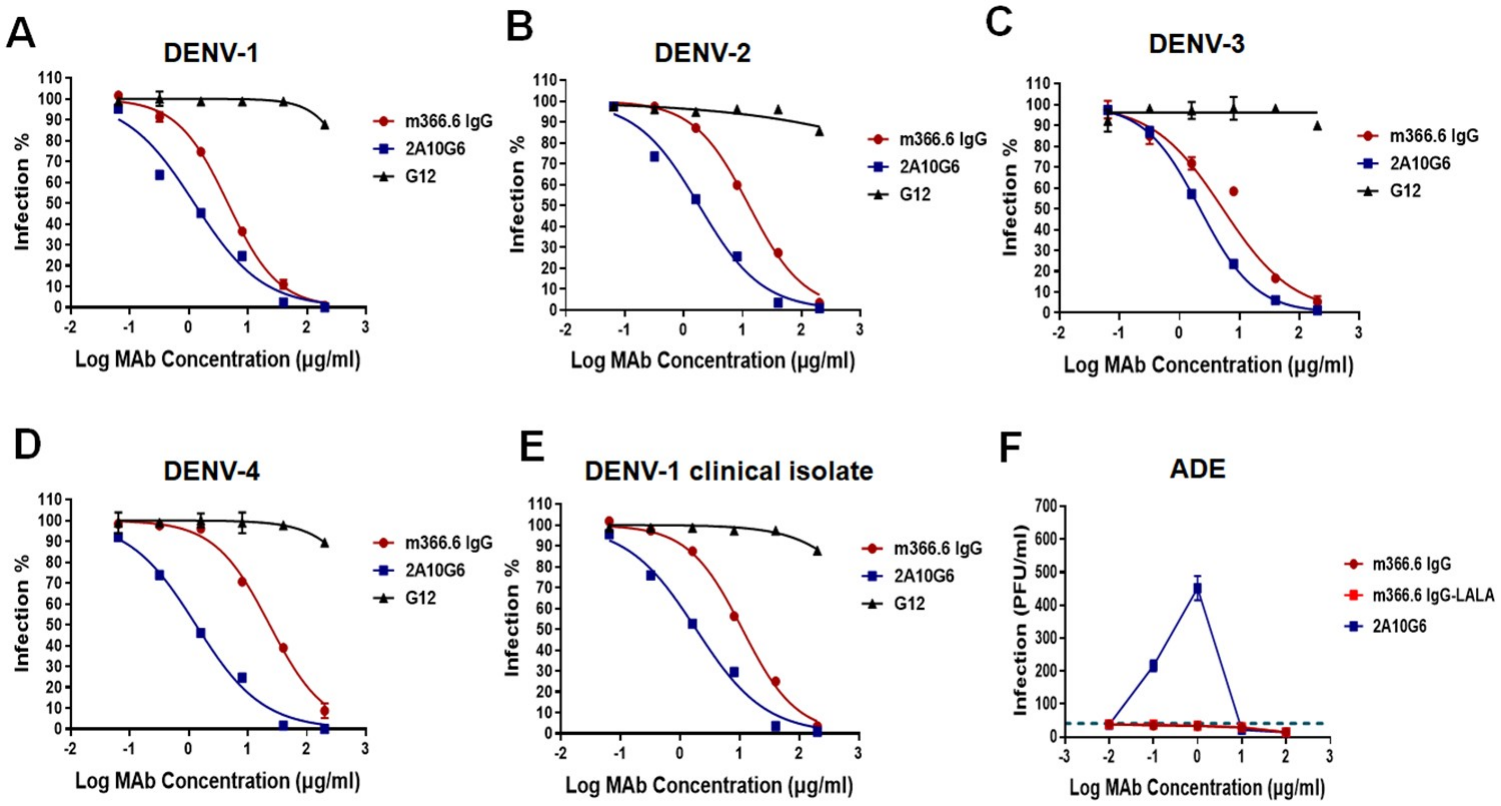
Neutralization and ADE effects of antibodies against different serotypes DENV. (A-E). DENV1-4 was mixed with 5-fold serial dilutions of mabs. Then neutralization activity was evaluated by plaque reduction assays in triplicates using BHK21 cells. Results were exhibited by percentage of plaques reduction and the independent neutralization experiments were performed in duplicate. (F) DENV-1 was incubate with 10-fold serial dilutions of mAbs before added to K562 cells. Virus in the supernatant of infected K562 cells was quantified in a plaque assay. The data were shown as means ± SD. The dotted line indicates the limit of detection. Results are representative of three independent experiments.

To further evaluate the neutralization breadth of m366.6 IgG against the four DENV serotypes, a standard plaque reduction neutralization assay (PRNT) on BHK-21 cells was performed using DENV1-4 live viruses, including DENV-1 128 (GenBank FJ176780), DENV-1 GZ01/2017 (S6 Fig, isolated from a DENV-1 infected patient in Guangzhou, China), DENV-2 43 (GenBank AF204178), DENV-3 80–2 (GenBank AF317645), and DENV-4 B5 (GenBank AF289029). An irrelevant human mAb G12 was used as the negative control [56], and 2A10G6, a broadly neutralizing mAb against all the four DENV serotypes, was used as the positive control [57,58]. As shown in Fig 3, m366.6 IgG could neutralize all the four DENV serotypes. The 50% neutralization titers (IC_50_) of m366.6 against DENV1-4 was 12.7, 4.57, 5.23, and 23.31 μg/ml respectively (Table 2).

**Table 2:** PRNT_50_ values of mAbs against DENV1-4.

IC_50_ (μg/ml)					
mAb	DENV-1 (128)	DENV-2 (43)	DENV-3 (80–2)	DENV-4 (B5)	DENV-1 (GZ01/2017)
**m366.6 IgG**	**12.74**	**4.57**	**5.23**	**23.31**	**12.56**
**2A10G6**	**1.82**	**1.23**	**2.19**	**1.31**	**1.84**
**G12**	**>200**	**>200**	**>200**	**>200**	**>200**

### ADE assay *in vitro*

We next used a well-established ADE assay to detect the *in vitro* ADE effect of m366.6 IgG. A mutated form of m366.6 IgG was also generated containing the leucine to alanine substitutions at positions 234 and 235 (m366.6 IgG-LALA), which lacked binding to Fcγ receptors. The ADE effects of DENV-1 or DENV-2 by m366.6 IgG, m366.6 IgG-LALA, as well as 2A10G6 were measured. Interestingly, neither m366.6 IgG nor m366.6 IgG-LALA presented any ADE effect against different serotypes of DENV (Fig 3F, S7 Fig). In contrast, potent ADE effects were observed for the DII-specific mAb 2A10G6. These results showed that m366.6 IgG is a DENV DIII-specific mAb without detectable ADE effect.

### Immunogenetic analysis

**Fig 4:**
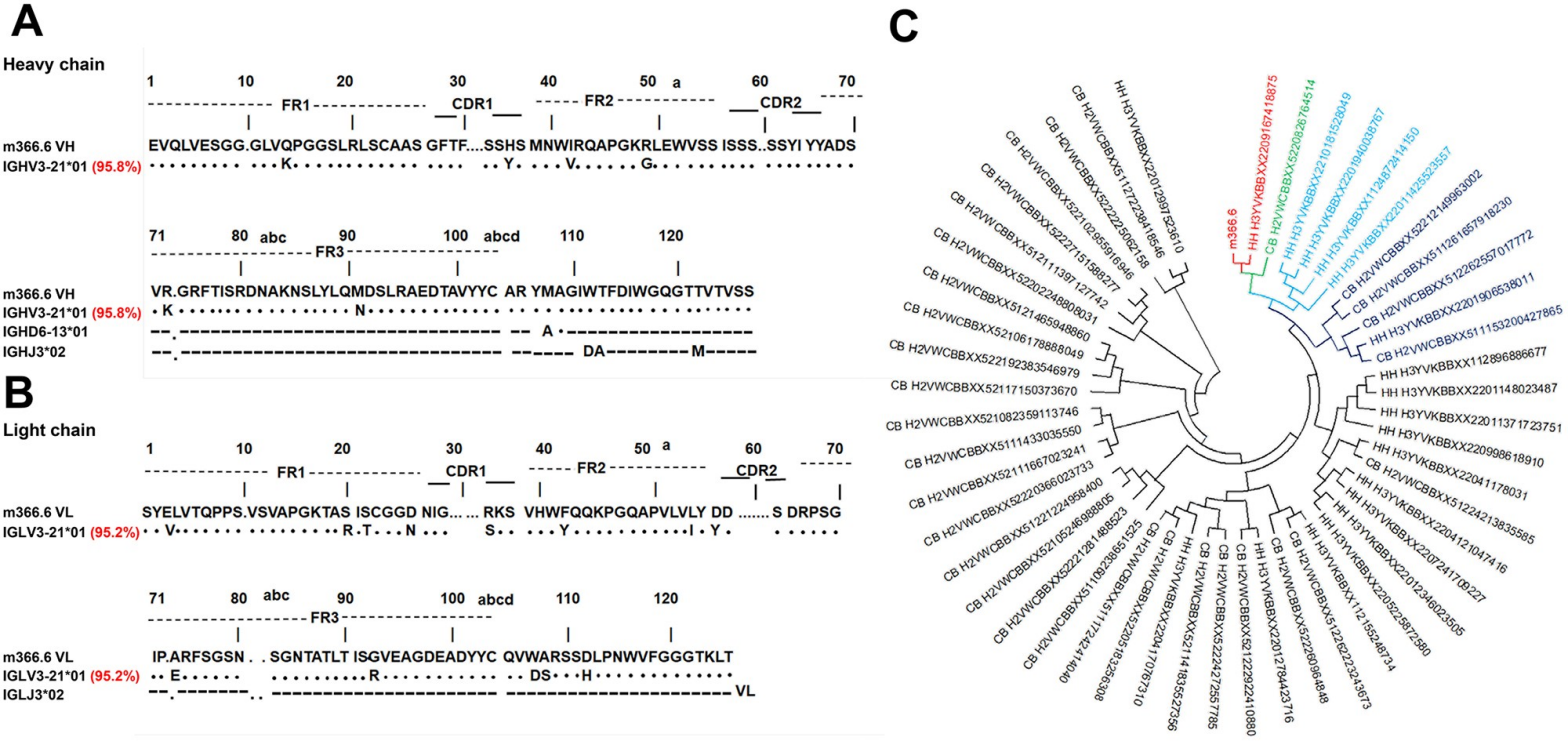
Immunogenetic analysis of m366.6. Immunogenetic analysis of the heavy- (A) and light-chain (B) variable regions of m366.6 using the IMGT tool. (C) Germline-rooted circular phylogenetic tree of m366.6-like antibody sequences found in IgM libraries derived from healthy human adults and neonates. The m366.6 and a sequence showed highest similarity to m366.6 were shown in red. Sequence ID started with CB represents sequence derived from the neonates, and that started with HH represents sequence derived from the healthy adults. The phylogenetic tree was constructed by the Neighbor-Joining method.

We further analyzed the sequences of mAbs using the IMGT tool to identify their closest VH and Vλ germline genes. The results indicated that m360.6 and m366.6 originated from different B-cell lineages (Table 3). The m360.6 VH gene was derived from the IGHV2-70 and the Vλ gene was from IGLV1-51. In contrast, the m366.6 VH gene was derived from the IGHV3-21 and the Vλ gene was from IGLV3-21. Interestingly, we found that the encoding genes of both m360.6 and m366.6 closely resembled their corresponding germline gene segments. Notably, m366.6 VH and Vλ gene shared 95.8% and 95.2% sequence identities with the IGHV3-21*01 and IGLV3-21*01 germlines respectively (Fig 4A and 4B). These results indicated that the mAb m366.6 is a germline-like antibody, which, in general, could show better drug properties and lower immunogenicity compared to somatically hypermutated antibodies [59].

**Table 3:** Genetic analysis of the heavy and light chain variable regions of DENV DIII-specific antibodies.

	mAb	Variable region	Variable region identity (%)	D	J	CDR3
**V_H_**	**m360.6**	HV2-70*10	**96.6%**	D1-1*01	J3*02	**VRTPYNWNDGPRGALDI**
	**m366.6**	HV3-21*01	**95.8%**	D6-19*01	J3*02	**ARYMAGIWTFDI**
**V_L_**	**m360.6**	LV1-51*01	**93.0%**		J2*01	**GAWDSRLSAVV**
	**m366.6**	LV3-21*01	**95.2%**		J3*02	**QVWARSSDLPNWV**

To further investigate the immunogenetic characteristics of m366.6-like antibodies, we analyzed in detail the IGHV3-21 recombination frequencies with specific IGHD and IGHJ genes families from naïve immunoglobulin M (IgM) repertoires of 33 health adult donors and neonatal IgM repertoires of 10 newborn babies, using next-generation sequencing data previously generated from our antibodyome studies [48]. By querying the m366.6 sequence from the IgM repertoires, 39 sequences were found to display m366.6-like V(D)J recombination from the genes IGHV-3-21, IGHD1, and IGHJ3 out of a total of 10,498,301 sequences from healthy adult IgM repertoires. In IgM repertoires of newborn babies, a similar recombination frequency was also observed, in which 111 sequences with m366.6-like V(D)J recombination were found from 5,617,227 sequences. Our analysis showed that IGHV3-21 is one of the most frequently used IGHV genes, and identified that many of those sequences sharing a significant degree of resemblance to m366.6 (Fig 4C). In brief, analysis of these data showed the potential of eliciting robust immune responses with the m366.6-like germline antibodies by vaccination.

### *In vivo* protection study in DENV1-4 infected mice

**Fig 5:**
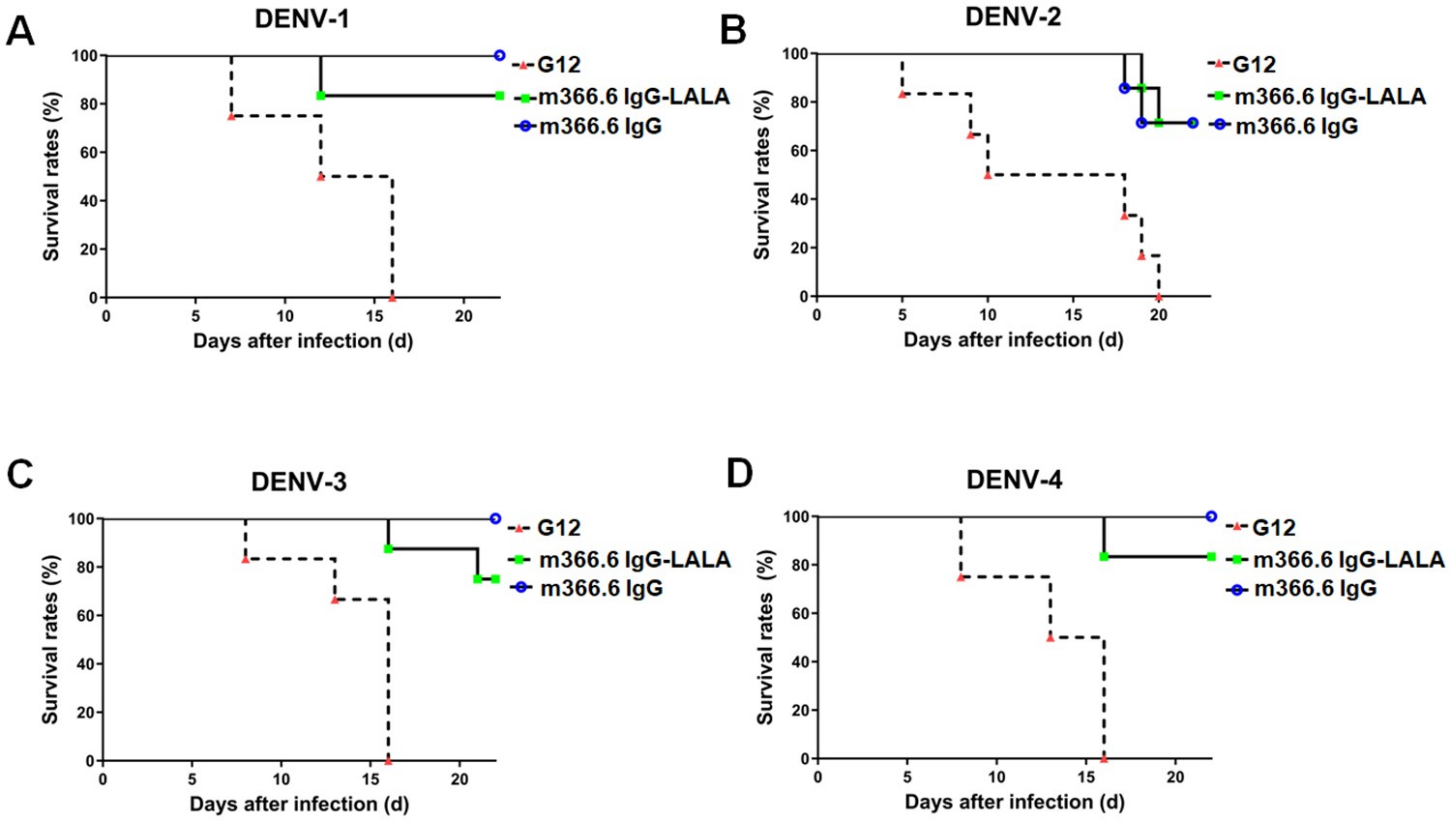
*In vivo* therapeutic efficacy of m366.6 against DENV1-4 infection. Groups of one day old suckling mice were administrated with 100 μg of mAbs 4 hours after challenge with 200 PFU of DENV-1 (A), DENV-2 (B), DENV-3 (C), and DENV-4 (D), respectively. Unrelated antibody G12 was used in the negative controls. The number of animals for each group ranged from 6 to 9. Kaplan-Meier survival curves were analyzed by the log-rank test and compared to curves of the controls.

To determine whether m366.6 can protect DENV infections *in vivo*, we firstly used a lethal DENV1-4 infection suckling mouse model. The mice were challenged with DENV1-4 at 200 PFU/mouse via intracranial injection. Four hours later, the mice were treated intracranial with a single dose (100 μg) of m366.6 IgG, m366.6 IgG-LALA mutant and G12 (unrelated antibody control). These animals were monitored for morbidity and mortality daily. As shown in Fig 5, all the mice in control groups died from DENV infection, and most of them died within the first two weeks of viral challenge. Interestingly, there was no significant difference in therapeutic efficacy against DENV1-4 infection between m366.6 and the LALA-mutated m366.6. The m366.6 IgG protected 100% DENV-1, DENV-3, DENV-4 and 83% DENV-2 infection whereas LALA-mutated m366.6 protected 83% DENV-1, DENV-4 and 67% DENV-2, DENV-4 respectively. Therefore, m366.6 has no detectable ADE as confirmed in both *in vitro* and *in vivo* experiments. We also used the AG129 (types-I and -II IFN receptor deficient) mice to test the therapeutic effect of m366.6 against DENV-2 (S8 Fig). The results showed that all the mice in the control antibody treatment group died while the survival rate of mice can reach 67% in m366.6 treatment group, indicating that the antibody can also protect the lethal infection of DENV-2 in AG129 mice. Taken together, these results indicated that m366.6 can protect DENV1-4 infections *in vivo*.

### Epitope mapping of m366.6

**Fig 6:**
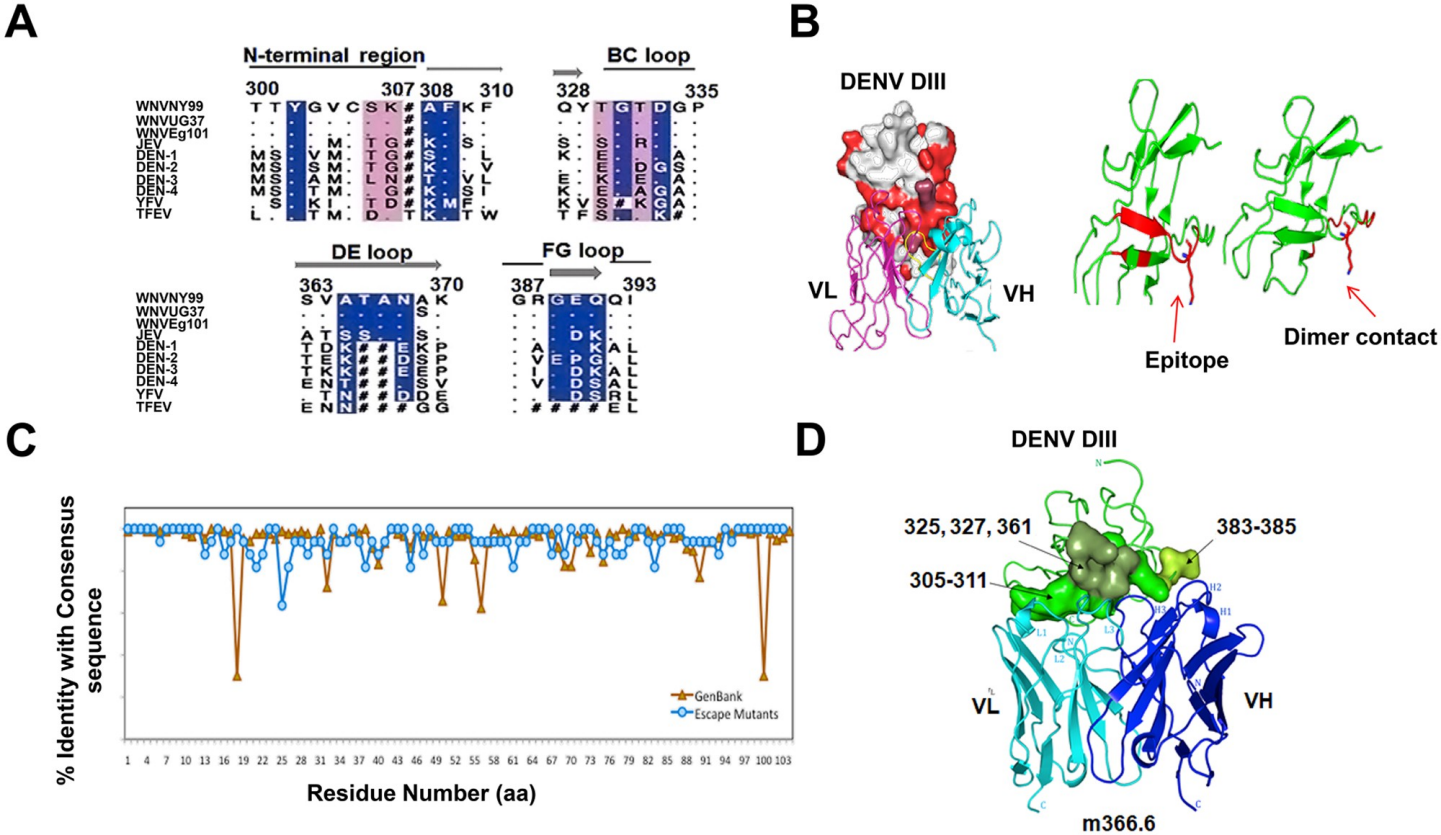
Epitope mapping of m366.6 on DENV DIII. (A) Sequence alignment of different flaviviruses between residues 300–393. (B) Epitope mapping on domain III.2 by sorting of DIII mutant library. The escape mutations were colored in red, and dominantly mapped to the lower region DENV DIII (PDB code: 2R69). The VH (colored in cyan) and VL (colored in magenta) of m366.6 were also shown to indicate the mapped epitope. (C) Sequence variation comparison of the DIII escape mutants and naturally isolated DENV DIIIs derived from GenBank. (D) Ribbon representation of DENV DIII-m366.6 scFv docked model in which experimentally identified epitope and contacting residues in the model were shown in green surface. The heavy and light chains are colored in blue and cyan, respectively, with CDRs labeled. Three distinct but structurally proximal epitope regions shown in different colors (green, smudge and lime) were labeled with residue numbers at putative locations in the model.

To map the epitope of the germline-like mAb m366.6 and identify in greater detail the structural basis of DENV neutralization, we employed multiple approaches (Fig 6).

Sequence alignment of different DENV genotypes and mapping of the conserved amino acid residues of DENV DIII showed that four serotypes DENV DIIIs amino acid residues were different from one another between amino acids 300–393 (Fig 6A). Subsequently, serotype 2 derived DIII consensus gene was randomly mutated to construct a yeast-displayed mutant library. Two rounds of sorting of those yeast cells showing expression on surface but lacking the binding to m366.6 was performed. A total of 35 binding escape mutants were aligned with the serotype 2 consensus protein sequence. Mutation frequency at each position was plotted against the residue position number. Similarly, 193 unique DIII sequences derived from naturally isolated serotype 2 dengue viruses from GeneBank were also aligned with the consensus sequence (Fig 6B and 6C). The superimposed profiles of the two set of sequences showed that many of the escaped mutations located in the well-conserved area, indicating the broad cross-reactivity of m366.6 to naturally isolated dengue viruses. Besides, the epitope mapping shows that the m366.6 epitope is at close to or partially overlaps the dimerization interface between domains II and III. These results may explain why m366.6 is a potent cross-reactivity antibody to all the four DENV serotypes.

Furthermore, computational docking of DENV DIII-m366.6 antibody complex was performed using ZDOCK method. We selected the three top scored docked complexes that contained the key residues identified from an experimental epitope mapping approach. One of the top scored docked models exhibited minimum clashes with appropriate protein interface parameters and was used to demonstrate the lactation the potential epitopes and their interactions with m366.6 antibody, which might shed light on the molecular mechanisms of broadly cross-reactive neutralization. Fig 6D showed the docking model of the DIII-m366.6 antibody complex in which these epitopes are highlighted in green surfaces. The docking model revealed a different orientation of antibody binding as compared to the DIII complex structure with Fab 1A1D-2 that was previously determined [35]. The epitope comprised of three structurally proximal regions, residues 305–311 in green, 325, 327 and 361 in dark green, and 383–385 at the C-terminal in lime. One of the key residues, K310, contacts the CDR-L1 of m366.6 which has a germline mutation. In Env-DIII-Fab-1A1D-2 complex structure, the residue K310 contacts the CDR-H1. The hydrophobic residues, Ile and Trp, of CDR-H3 contact the center part of the epitope, and other loops H1, H2, L2 and L3 also involve in the binding. The surface area of the interface between DIII and m366.6 antibody in the model complex is 716 Å^2^, a typical of antibody-antigen interactions. There are six hydrogen bonds likely to form and no salt bridges at the interface. In brief, the binding regions of the m366.6 may be close to or partially overlaps the dimerization interface between domains II and III, which might indicate the broad cross-reactivity of m366.6 to the four serotypes of DENV.

## Discussion

Dengue is a disease with a complex immune response orchestrated by host cells partially due to the presence of four serotypes of DENV. Importantly, after a primary DENV infection, one can be protected against or aggravate of a secondary infection with a different serotype, which bring many difficulties to develop an effective vaccine. Thus, it is very urgent to develop an effective and cross-reactive antiviral therapy against DENV infection.

Monoclonal antibodies (mAbs) are of growing importance for protective and pathogenic immune responses to viruses. At present, there are many therapeutic antibodies to treat viral infections under development, such as antibodies for HIV-1, SARS-CoV, MERS-CoV, Nipah and Hendra viruses, and H7N9 influenza virus [48,50,60–66]. Fortunately, screening antibodies from the large naïve libraries has enabled the rapid development of high-affinity human mAbs, especially for the rapid response to the outbreak of emerging viruses and diseases. We recently successfully identified two human germline-like mAbs against MERS-CoV and H7N9 influenza virus from the naïve library, named m336 and m826, respectively [48,50]. They both can naturally exist with very low level of somatic hypermutation in the naïve library with which they have potent binding activity against the envelop proteins of MERS-CoV and H7N9 influenza virus. Most importantly, m336 and m826 all showed highly therapeutic effective in the animal models. Therefore, the naïve library screening can be quickly used to isolate germline-like antibodies that effectively bind to complex protein targets like those in DENV viruses.

How to increase the neutralization breadth is a key issue in developing anti-DENV antibodies. Previous studies revealed two classes of broadly neutralizing antibodies to flaviviruses, including antibodies targeting the conserved epitopes in DII or DIII [16–18,20]. While the conserved fusion loop epitope (FLE) in DII is the immunodominant epitope in E protein, unfortunately, this epitope frequently induced poorly neutralizing and strongly infection-enhancing antibodies via ADE [18–20]. Therefore, DIII represents the ideal target for neutralizing antibodies. In this study, we applied a highly efficient yeast-display-based sorting strategy by using the highly diverse DENV DIIIs as antigen and the competitive sorting technique. By applying this method, we quickly and efficiently identified two human germline-like broad-spectrum anti-DENV mAbs (m360 and m366) from the naïve scFv yeast library using the DIII antigen that make them as promising candidate therapeutics as well as the template for vaccine development. Another class of highly efficient broadly neutralizing antibodies that target the envelope dimer epitopes (EDE) from the secondary acute DENV infection plasmablasts has been identified by Dejnirattisai *et al*. [67]. These antibodies may especially get through with high somatic mutations from the secondary virus infection. Compared with the highly somatically mutated antibodies, germline-like antibodies typically have better safety and drug-related property [59]. Importantly, the Hendra and Nipah antibody m102.4 is a near germline antibody and exhibited a very good drugability, which was from a similar library that was also used to isolate our m366 and m366-like antibodies. m102.4 was successful as a candidate therapeutic mAb in animal models and was also completed the phase I clinical trial (ACTRN12615000395538) without side effects [64]. To further improve the affinity of m366 with the four serotypes DENV DIIIs, we subjected m366 to affinity maturation process, and named it as m366.6. Subsequently, we analyzed m366.6 sequence using the IMGT tool to identify its closest VH and Vλ germline genes. Interestingly, we found that m366.6 is still a germline-like antibody although it went through the mutation process *in vitro*, with over 95% identities of its VH and Vλ genes to the IGHV3-21*01 and IGLV3-21*01 germline respectively.

In order to evaluate the neutralization effect of the m366.6 IgG, we used a standard plaque reduction neutralization with BHK21 cells to measure DENV infection and neutralization. The m366.6 IgG showed broadly neutralization towards the four serotypes DENV as well as a recent DENV isolate from clinical samples. More importantly, m366.6 did not present any ADE effects in different serotypes of DENV. The *in vivo* study results demonstrated the therapeutic potential of m366.6 against severe DENV1-4 infections. In brief, the m366.6 could neutralize the four serotypes DENV *in vitro* and protect the DENV infection mouse model *in vivo* without detectable ADE effects. We therefore expect that m366.6 has a likeness drugability of m102.4 and could be developed as a candidate therapeutic in the future.

We have also localized the m366.6 epitope by using a combination of computational structural modeling, display-based antigen mutagenesis, and sequence-based analysis of mutants. The epitope appears to overlap with the epitope previously explored as targets for cross-reactive murine mAbs and close to or partially overlaps the dimerization interface between domains II and III. This further indicates that this epitope could be an important component of vaccine immunogens intended to elicit cross-reactive neutralizing antibodies. In progress are our experiments to crystallize the complex of m366.6 with DENV DIII that would allow precise determination of the m366.6 epitope.

The major result of this study is the identification of a germline-like human mAb, m366.6, from a naïve yeast antibody library which binds with high (picomolar) affinity to DIIIs from all serotypes and neutralizes the four DENV serotypes. There are two major implications from this finding: 1) m366.6 is a potential candidate therapeutic which could be further developed in preclinical and clinical settings. 2) the epitope of the germline-like mAb m366.6 could guide the design of effective candidate vaccine immunogens capable of eliciting m366.6 and/or m366.6-like antibodies.

## Materials and methods

### Cell lines and viruses

BHK21 cells were cultivated in Dulbecco’s Modified Eagle Medium (DMEM) supplemented with 10% fetal bovine serum (FBS) (Biowest). Mosquito cells C6/36 were cultured in RPMI-1640 medium supplemented with 10% FBS. All cells were maintained in a humidified atmosphere of 5% CO_2_ at 37°C incubator, except for C6/36 cells, which were cultivated at 28°C. DENV-1 128 (GenBank FJ176780), DENV-1 GZ01/2017 (isolated from DENV-1 infected patient in Guangzhou), DENV-2 43 (GenBank AF204178), DENV-3 80–2 (GenBank AF317645), and DENV-4 B5 (GenBank AF289029) were propagated in C6/36 cells by using RPMI 1640 medium and the titers were measured by standard plaque forming assay in BHK21 cells.

### Protein expression and purification

DENV DIII genes from all 4 serotypes were synthesized by Genescript, Inc (Nanjing, China), fused with IgG1 Fc and a C-terminal Avi-tag, and cloned into pSecTag expression vector. The DIII.3 (serotype 3) K310E mutant was generated through overlapping PCR. For the conversion of IgG1 from scFv, the heavy and light chains of scFv were amplified and recloned into the PTT-IgG1 vector. The plasmids were transfected into Expi293 cells (Thermo Fisher) for transient expression, and purified using protein G column (GE Healthcare, Piscataway, NJ) according to the manufacturer’s instructions. The purified protein was biotinylated by mixing with biotinylation reagents in PBS for 30 min on ice, according to the manufacturer’s instructions (Pierce).

### Antibody screening from yeast library

A large yeast-displayed scFv library was used for antibody screening, and the screening protocols were essentially carried out as described previously [55]. Briefly, 10 μg of binotinylated DIII.3-Fc and 10^10^ cells of the initial naïve library were mixed and washed by PBSA, and incubated with 100 μl streptavidin conjugated microbeads (Miltenyi Biotec, Auburn, CA) before loading onto the autoMACS system (Miltenyi Biotec) for sorting. After three rounds of sorting, the downsized library was further sorted against binotinylated DIII.3-Fc (1 μg/ml) but also using unbiotinylated K310E mutant (1 μg/ml) as the competitor. The cells were stained by the addition of mouse anti-c-Myc antibody (Roche), Alexa-488 conjugated goat-anti-mouse antibody (Invitrogen), and PE-conjugated streptavidin (Invitrogen) for sorting on a FACSAria II cell sorter (BD Biocsiences, San Jose, CA) to isolate the positive binders. The plasmids of the positive clones were prepared by using Zyppy Plasmid Miniprep Kit according to the manufacturer’s instructions (Zymo Research).

### Mutant library construction through error-prone and DNA shuffling PCR

To generate the m360 scFv and m366 scFv mutant libraries, random mutagenesis of the scFv genes were performed through error-prone PCR by using a GeneMorph II kit (Stratagene) following the manufacturer’s instructions with minor modifications. To further diversify the mutation profile, 3 uM of each of the two nucleotide analogues (8-oxo-deoxyguanosine triphosphate and 2'-deoxy-p-nucleoside-5'-triphosphate) was mixed in the PCR reaction mixture. For the second and third cycle library constructions, an extra step of DNA shuffling PCR was inserted into the regular PCR cycles to combine the beneficial mutations obtained from previous maturation process. DNA shuffling PCR step was performed as following: 20 cycles of denature at 94 °C for 15 seconds followed by annealing/extension at 68°C for 1 second on the Bio-Rad MyCycler.

### Affinity determination by Surface Plasmon Resonance

Binding affinities of m360 scFv, m366 scFv, m360.6 scFv, and m366.6 scFv to the 4 DENV DIIIs were analyzed by surface plasmon resonance technology using a Biacore X100 instrument (GE healthcare). The antibodies were covalently immobilized onto a sensor chip (CM5) using carbodiimide coupling chemistry. A control reference surface was prepared for nonspecific binding and refractive index changes. For analysis of the kinetics of interactions, varying concentrations of antigens were injected at flow rate of 30 μl/min using running buffer containing 10mM HEPES, 150 mM NaCl, 3 mM EDTA, and 0.05% Surfactant P-20 (pH 7.4). The association and dissociation phase data were fitted simultaneously to a 1:1 Langumir global model by using the nonlinear data analysis program BIAevaluation 3.2. All the experiments were done at 25°C.

### DENV PRNT

Neutralizing activity of mAbs was measured using a standard plaque reduction neutralization with BHK21 cells as previously described [57]. Briefly, 5-fold serial dilutions of mAbs were added to approximately 200 PFU of a variety of dengue virus strains and incubated for 1 h at 37°C. Then, the mixture was added to BHK21 cell monolayers in a 12-well plate in duplicate and incubated for 1 h at 37 °C. The mixture was removed, and 1 ml of 1.0% (w/v) LMP agarose (Promega) in DMEM plus 4% (v/v) FBS was layered onto the infected cells. After further incubation at 37 °C for 4 days, the wells were stained with 1% (w/v) crystal violet dissolved in 4% (v/v) formaldehyde to visualize the plaques. PRNT_50_ values were determined using non-linear regression analysis. PRNT_50_ data were calculated by doing a non-linear regression analysis using Sigmaplot (Version 9.01, Systat Software, Inc., CA) as previously described [57].

### DENV RVP neutralization assay

DENV RVPs from all four serotypes were pre-incubated with an equal volume of serially diluted antibodies (25 μg/ml to 0.0012 μg/ml pre-dilution or 12.5 μg/ml to 0.0006 μg/ml pre-dilution, as measured based on the dilution of antibody prior to combining with RVPs) in DMEM infection media for 1 h at room temperature and transferred to wells of a 96-well plate. An equal volume of DENV RVPs were added to each well followed by slow agitation for 1 h at room temperature. BHK DC-SIGN cells were added to each well at a density of 30,000 cells per well followed by incubation at 37°C in 5% CO_2_ for 48 h. Cells were subsequently fixed in lysed and analyzed for luminescent reporter expression using the Wallac Victor. The percent infection for each concentration of mAb or serum was calculated, and the raw data was expressed as percent infection versus log_10_ of the mAb concentration or the reciprocal serum dilution. The data were fit to a sigmoidal dose-response curve using Prism (GraphPad Software, La Jolla, CA) to determine the titer of antibody that achieved a 50% reduction in infection. Maximum infection was determined in the absence of antibodies.

### DENV ADE assay

The *in vitro* ADE assay was performed using K562 cells [57]. Briefly, serial 10-fold dilutions of antibodies under concentrations ranging from 100 to 0.01 μg/ml were mixed with DENV-1 or DENV-2, and incubated for 1 h at 37 °C. Mixtures were then added to 2×10^5^ K562 cells at multiplicity of infection of 0.1~0.25 for 2 h in 24-well plates. The cells were subsequently washed 3 times with serum free RPMI-1640 medium. After collecting cells by centrifugation, the cell pellets were re-suspended with RPMI-1640 medium containing 2% FBS and added to 24-well plates, then incubated for 4 days at 37 °C with 5% CO_2_. The titer of viruses in the supernatant was then measured using a plaque assay. The ADE effect was calculated as different viral yields in the supernatant after infection in the presence of the added antibodies.

### Epitope mapping of m366.6 through DIII mutant library sorting

The epitope mapping of m366.6 was performed using previously described protocols [55]. Briefly, random mutagenesis of the DENV DIII.2 gene was performed using a GeneMorph II kit (Stratagene). As described above, the yeast-displayed mutant library was mixed with biotinylated m366.6 scFv-Fc, washed, and stained by mouse anti-c-Myc antibody (Roche), Alexa-488 conjugated goat-anti-mouse antibody (Invitrogen), and PE-conjugated streptavidin (Invitrogen). After two rounds of sorting on a FACSAria II cell sorter (BD Biocsiences, San Jose, CA), the sorted cells were amplified and their plasmids were prepared and sequenced.

### Computational docking of the DIII-m366.6 antibody complex

Homology modeling of variable regions of heavy (V_H_) and light (V_L_) chains for m366.6 scFv antibody was carried out using the SWISS-MODEL workspace [68] by selecting the closest template structures (PDB codes: 3QOS for heavy chain and 2DD8 for light chain), whose sequence similarities were 92% and 87% respectively. The V_H_-V_L_ orientation of m366.6 scFv structure was assigned similar with one of the templates (PDB code: 2DD8) that showed minimum steric clash for creating the final m336.6 scFv model. The crystal structure of DENV DIII serotype 2 (PDB code: 2R29) was used for docking with the modeled scFv antibody m366.6. Docking of scFv m336.6 to the dengue Env-III was performed by ZDOCK server (http://zdock.bu.edu) that uses a fast Fourier transform (FFT)-based rigid-body protein docking algorithm with scoring functions combining pairwise shape complementarity, desolvation and electrostatic energies. Based on the escape mutants that led to the loss of epitopes and available crystal structure of DENV DIII, we selected a list of residues as biological constrains, 307, 309, 310, 311, 327, 361 and 383, on the surface of Env-DIII as potential contacting residues for docking constraints. Similarly, one or two residues from each of CDR-H1, H3 and L3 loops were chosen at the docking interface. CDR-H1 and H3 loops had dominant hydrophobic residues whereas CDR-L1 had a germline mutation, and they all had high antigen-contacting propensities [69]. Results from the top 2000 ZDOCK predictions were filtered using the user-defined residues and a 6 angstrom distance cutoff. Three predicted complexes were only kept as all residues selected come together at the interface and were further examined by PDBePISA (Protein Interfaces, Surfaces and Assemblies). PyMOL was used for the analysis of docked model and graphical illustration [70].

### *In vivo* therapeutic experiments

The suckling mice were purchased from B&K Universal Group Limited (Shanghai, China) and housed under specific pathogen-free conditions at the animal facilities of the Shanghai Public Health Clinical Center, Fudan University (Shanghai, China). Before infection, the mice were transferred to the Animal Biosafety Level 2 (BSL-2) Laboratory (Shanghai, China). One day mice were used for all experiments. All mice were intracerebrally injected with 200 PFU of DENV1-4. At 4 h post infection, mice were passively transferred a single dose of 100 μg antibody m366.6 IgG, m366.6 IgG LALA mutant or G12 IgG as the negative control via intracerebrally injection. Survival rates and disease sings were monitored daily. The AG129 mice (type I and type II interferon receptor-deficient) were purchased from B&K Universal Group Limited (Shanghai, China) and housed under specific pathogen-free conditions at the animal facilities of the Shanghai Public Health Clinical Center, Fudan University (Shanghai, China). Before infection, the mice were transferred to the BSL-2 Laboratory (Shanghai, China). Groups of mixed-sex 4- to 6-week-old mice were used for all experiments. All mice were intraperitoneally injected with 2x10^6^ PFU of DENV-2 in a volume of 200 μL. At 16 h post infection, mice were passively transferred a single dose of 500 μg antibody m366.6 IgG-LALA, or G12 antibody as the control via i.p. injection. Survival rates, weight loss, and disease sings were monitored daily.

### Ethics statement

Specific-pathogen-free AG129 mice (4–6 weeks old) and suckling mice were used for all experiments. All experimental protocols were reviewed and approved by the institutional committee of Fudan University (Permit Number: 2018-A056-02) in accordance with the Guideline for Ethical Review of Animal Welfare (GB/T 35892–2018) of the Chinese National Health and Medical Research Council (NHMRC).

## Supporting information

S1 Fig Binding of m360 to DENV DIIIs from the four serotypes measured by Biacore. The m360 was immobilized onto a CM5 chip, and the analytes consisted of serial dilution of DIII from DENV-1 (A), DENV-2 (B), DENV-3 (C), or DENV-4 (D). Binding kinetics was fitted using a 1:1 Langumir binding model by BIAevaluation 3.2 software.
(TIF)

S2 Fig Binding of m366 to DENV DIIIs from the four serotypes measured by Biacore. The m366 was immobilized onto a CM5 chip, and the analytes consisted of serial dilution of DIII from DENV-1 (A), DENV-2 (B), DENV-3 (C), or DENV-4 (D). Binding kinetics was fitted using a 1:1 Langumir binding model by BIAevaluation 3.2 software.
(TIF)

S3 Fig Binding of m360.6 to DENV DIIIs from the four serotypes measured by Biacore. The m360.6 was immobilized onto a CM5 chip, and the analytes consisted of serial dilution of DIII from DENV-1 (A), DENV-2 (B), DENV-3 (C), or DENV-4 (D). Binding kinetics was fitted using a 1:1 Langumir binding model by BIAevaluation 3.2 software.
(TIF)

S4 Fig Binding of m366.6 to DENV DIII, ZIKV DIII, gp140 and PDL1 proteins measured by ELISA. (TIF)

S5 Fig Broad neutralization of the four DENV serotypes by m360.6 and m366.6. (A-D) Infectivity of DENV RVPs for all four serotypes. RVPs for DENV-1 (WestPac), DENV-2 (S16803), DENV-3 (CH53489) or DENV-4 (TVP360) were serially diluted in DMEM. BHK DC-SIGN cells were added and cells were cultured for 72 h. The cells were then lysed and examined for reporter expression. The independent neutralization experiments were performed in duplicate.
(TIF)

S6 Fig The complete sequence of DENV-1 GZ01/2017, a dengue virus isolated from a DENV-1 infected patient in Guangzhou, China. (TIF)

S7 Fig ADE activity of antibodies against DENV-2. DENV-2 was incubate with 10-fold serial dilutions of mAbs before added to K562 cells. Virus in the supernatant of infected K562 cells was quantified in a plaque assay. The data were shown as means ± SD. The dotted line indicates the limit of detection.
(TIF)

S8 Fig In vivo therapeutic efficacy of m366.6 against DENV-2 infection. For therapeutic efficacy study, AG129 mice were treated intraperitoneally with and m366.6 IgG-LALA 16 h after viral challenge with 2x10^6^ PFU of DENV-2, and were monitored daily for 12 days for the accumulated mortality (n = 6 per group). Unrelated antibody G12 was used for the control group.
(TIF)
