# Supplementary figures and images for: A broadly neutralizing germline-like human monoclonal antibody against dengue virus envelope domain III
Source: PLoS Pathog. 2019 Jun 26;15(6):e1007836. doi: 10.1371/journal.ppat.1007836 (PMC6615639; doi:10.1371/journal.ppat.1007836)

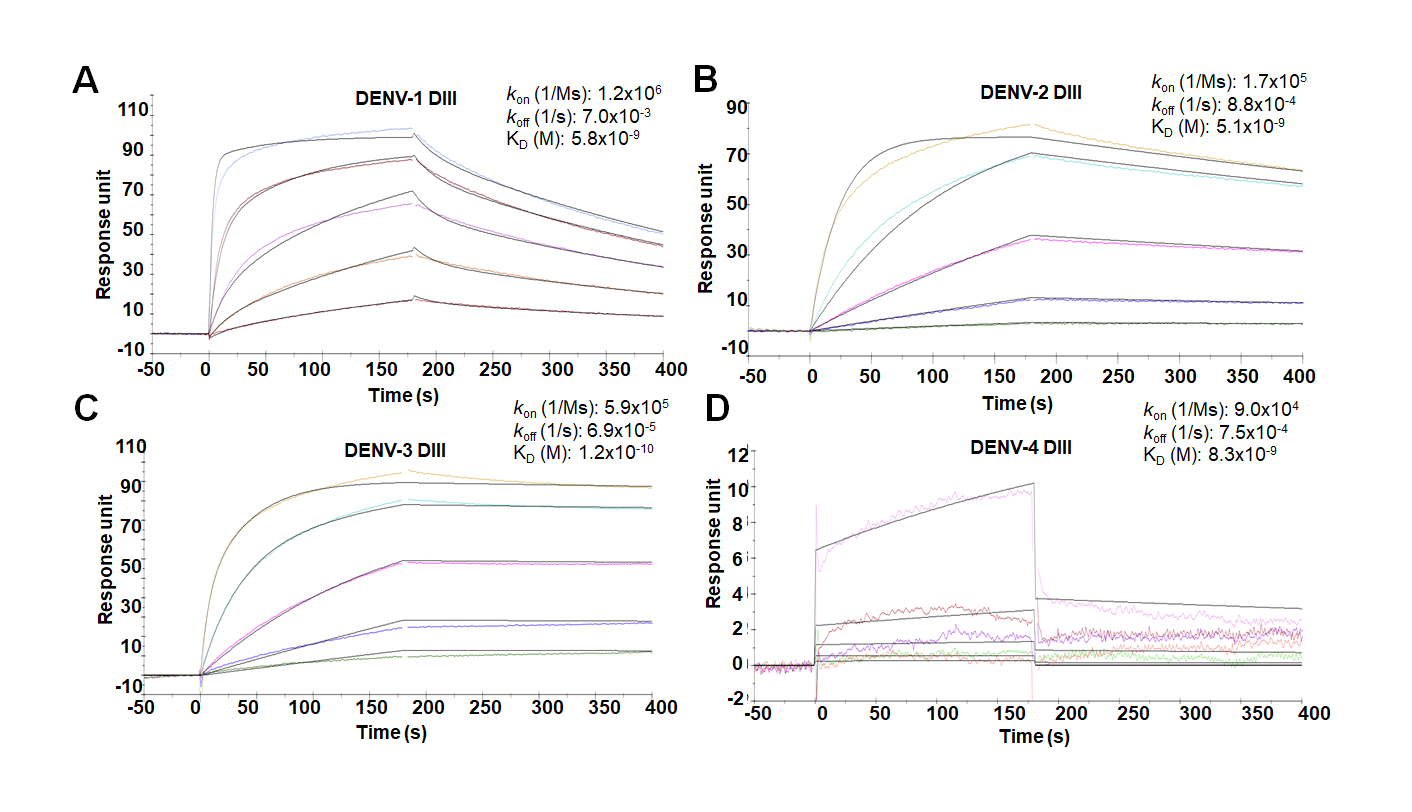

Supplement: S1 Fig — The m360 was immobilized onto a CM5 chip, and the analytes consisted of serial dilution of DIII from DENV-1 (A), DENV-2 (B), DENV-3 (C), or DENV-4 (D). Binding kinetics was fitted using a 1:1 Langumir binding model by BIAevaluation 3.2 software. (TIF) [file ppat.1007836.s001.tif]

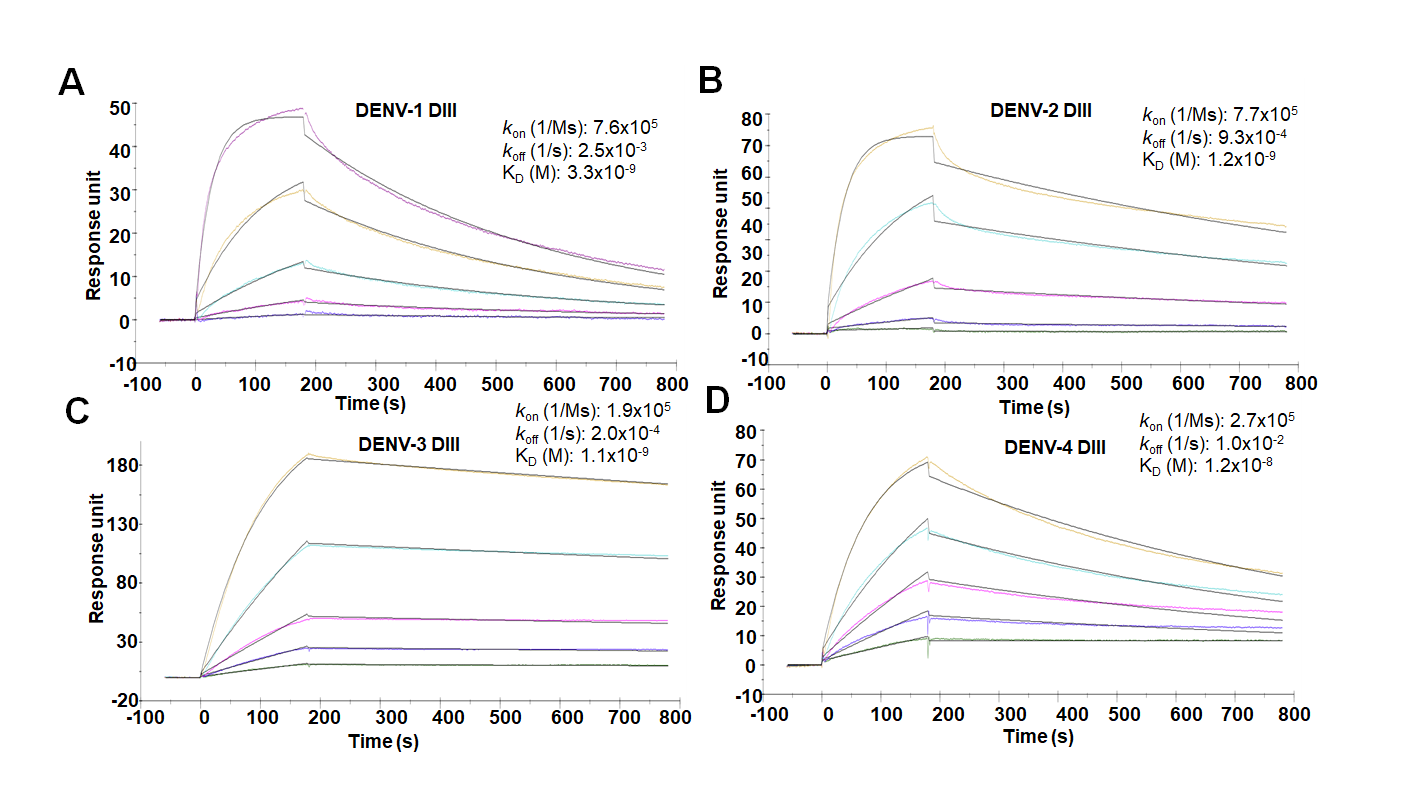

Supplement: S2 Fig — The m366 was immobilized onto a CM5 chip, and the analytes consisted of serial dilution of DIII from DENV-1 (A), DENV-2 (B), DENV-3 (C), or DENV-4 (D). Binding kinetics was fitted using a 1:1 Langumir binding model by BIAevaluation 3.2 software. (TIF) [file ppat.1007836.s002.tif]

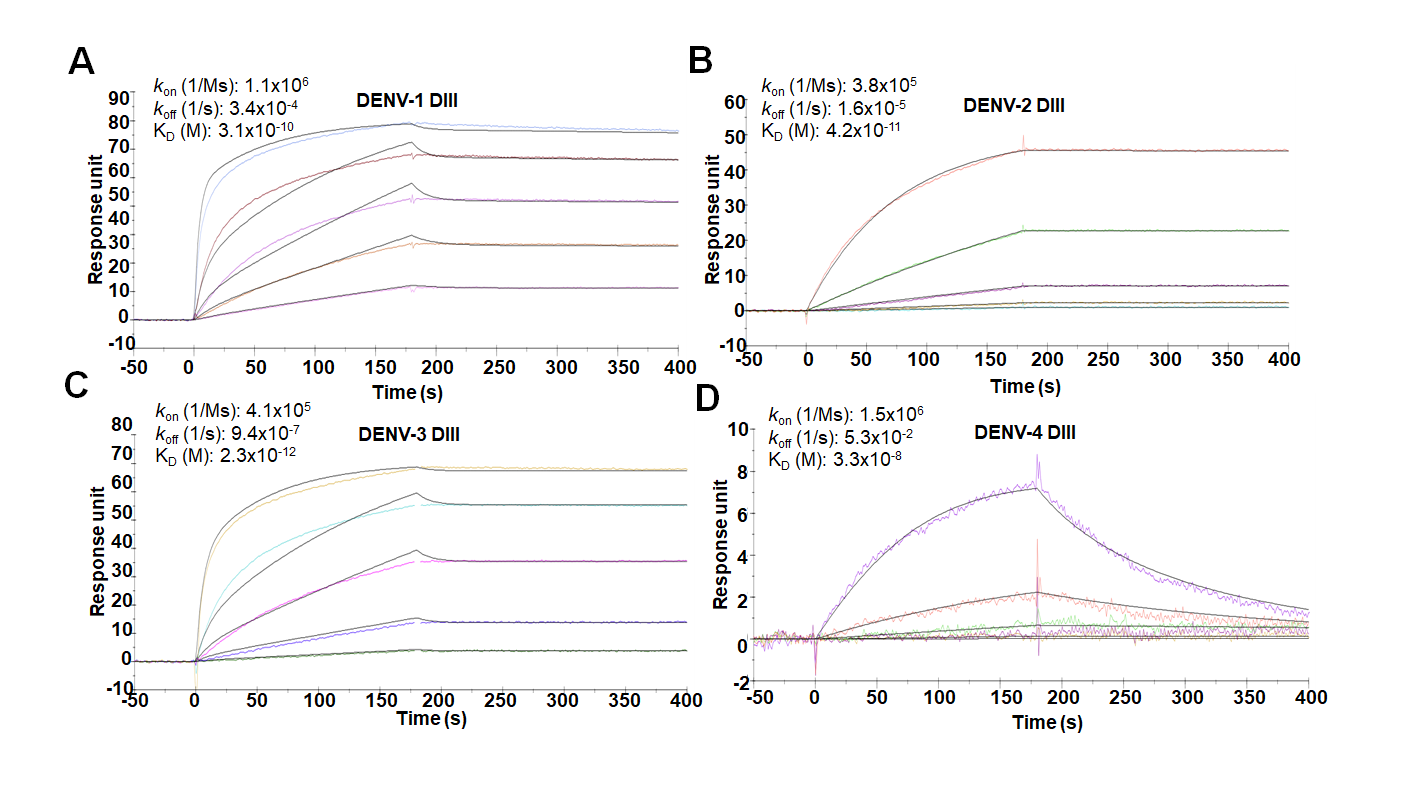

Supplement: S3 Fig — The m360.6 was immobilized onto a CM5 chip, and the analytes consisted of serial dilution of DIII from DENV-1 (A), DENV-2 (B), DENV-3 (C), or DENV-4 (D). Binding kinetics was fitted using a 1:1 Langumir binding model by BIAevaluation 3.2 software. (TIF) [file ppat.1007836.s003.tif]

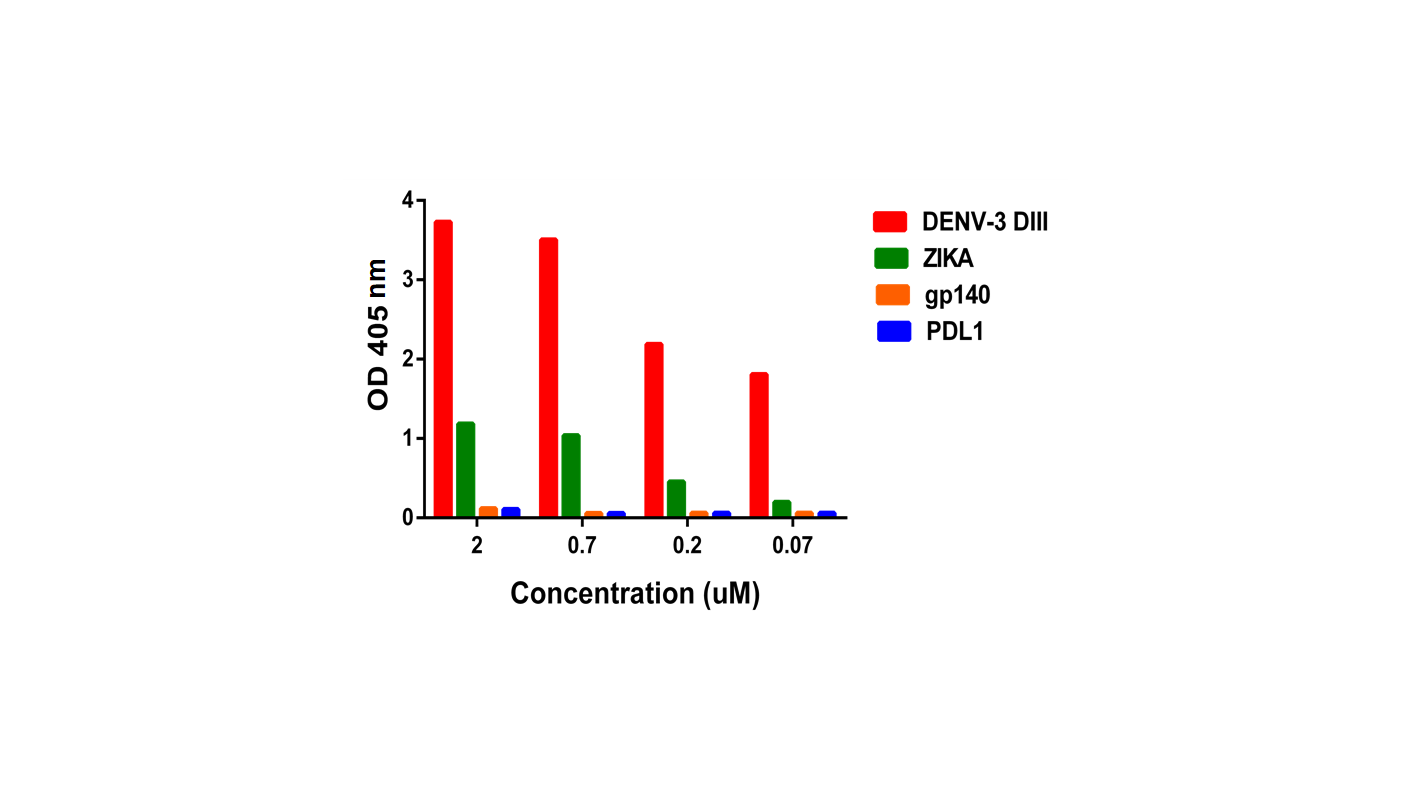

Supplement: S4 Fig — (TIF) [file ppat.1007836.s004.tif]

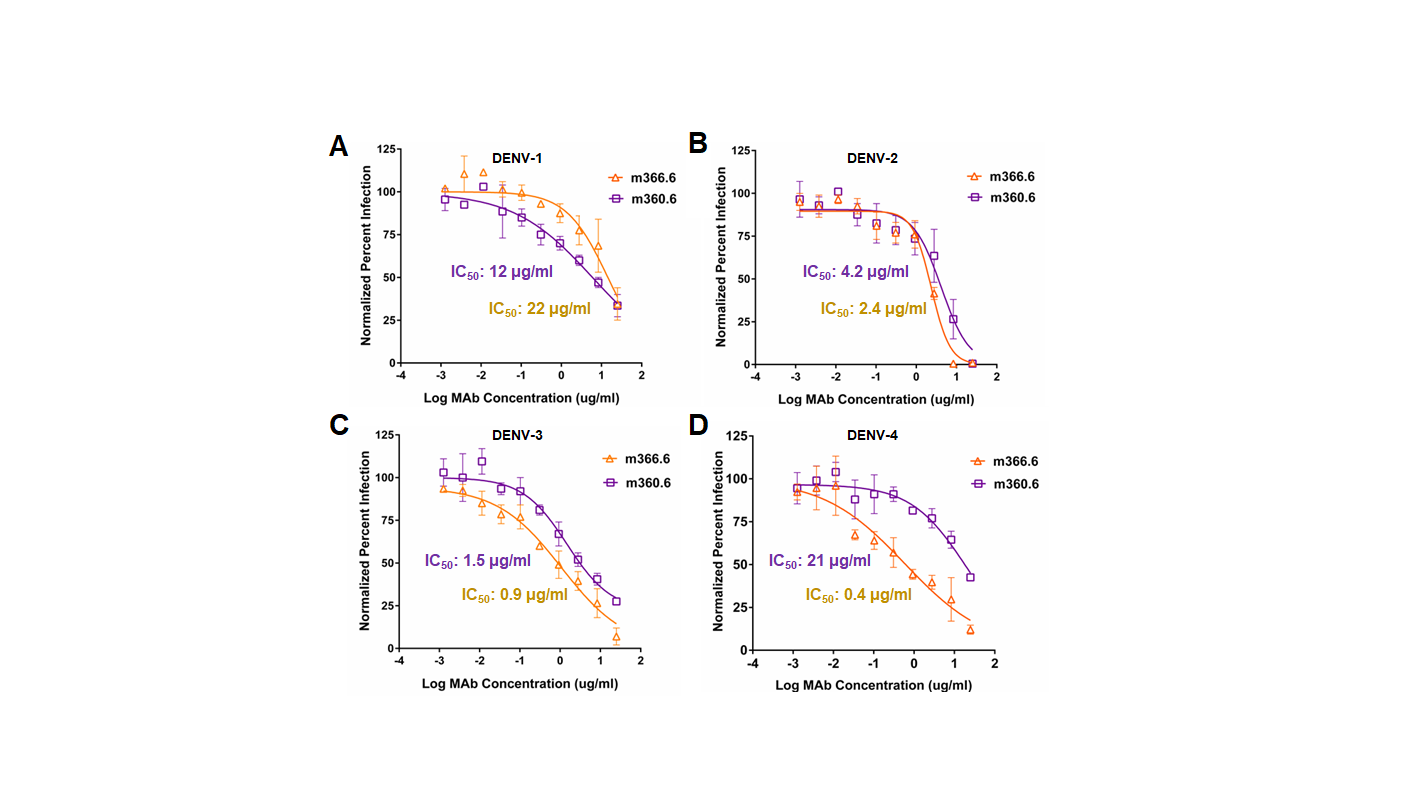

Supplement: S5 Fig — (A-D) Infectivity of DENV RVPs for all four serotypes. RVPs for DENV-1 (WestPac), DENV-2 (S16803), DENV-3 (CH53489) or DENV-4 (TVP360) were serially diluted in DMEM. BHK DC-SIGN cells were added and cells were cultured for 72 h. The cells were then lysed and examined for reporter expression. The independent neutralization experiments were performed in duplicate. (TIF) [file ppat.1007836.s005.tif]

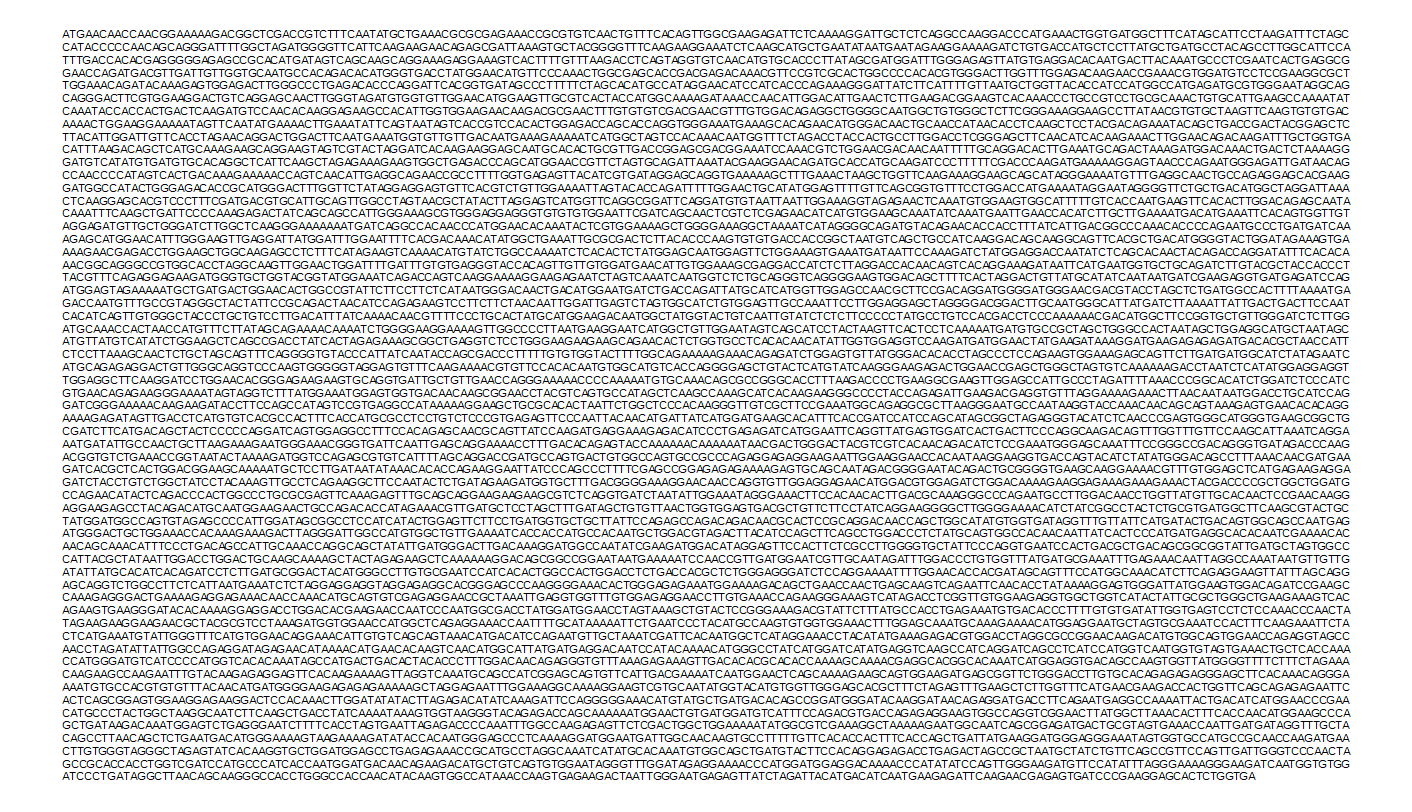

Supplement: S6 Fig — (TIF) [file ppat.1007836.s006.tif]

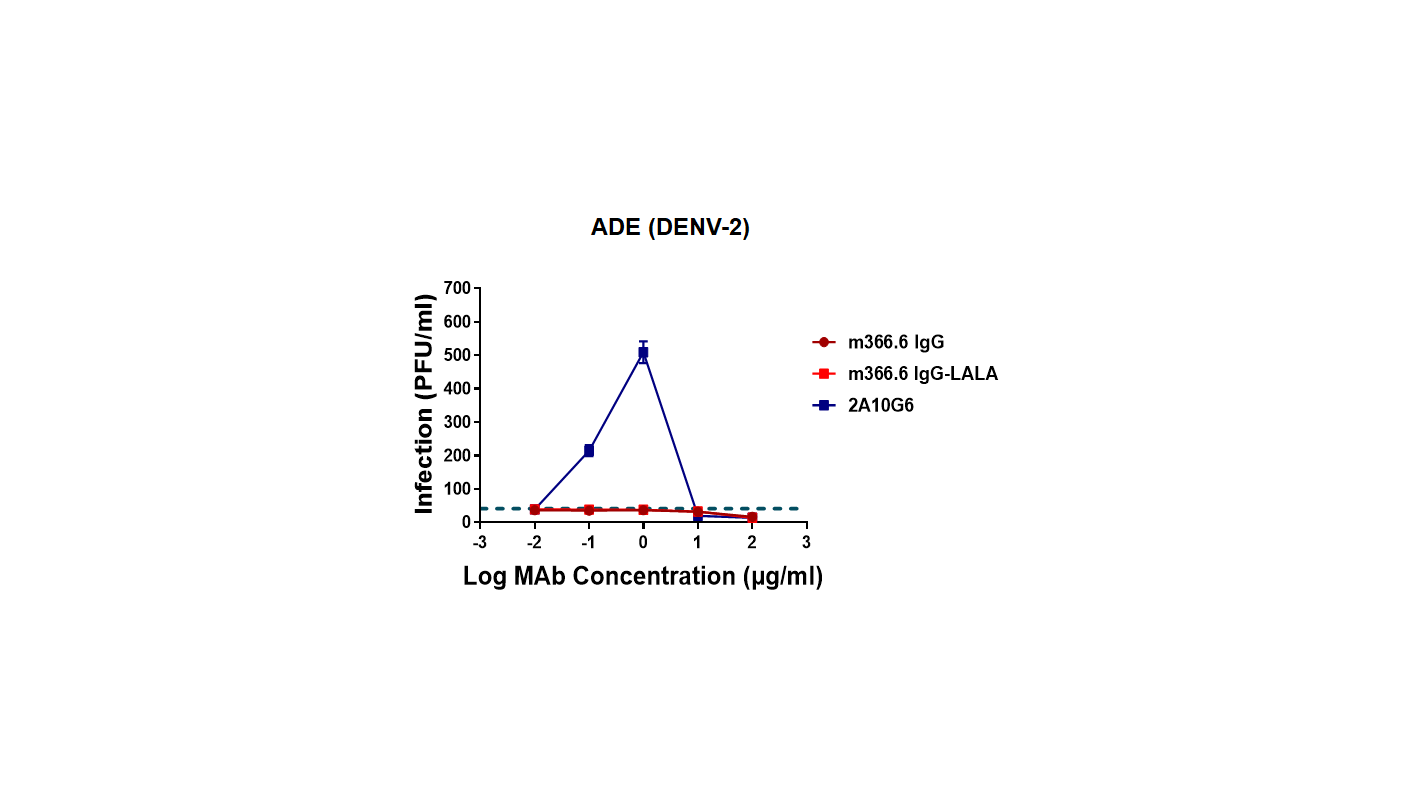

Supplement: S7 Fig — DENV-2 was incubate with 10-fold serial dilutions of mAbs before added to K562 cells. Virus in the supernatant of infected K562 cells was quantified in a plaque assay. The data were shown as means ± SD. The dotted line indicates the limit of detection. (TIF) [file ppat.1007836.s007.tif]

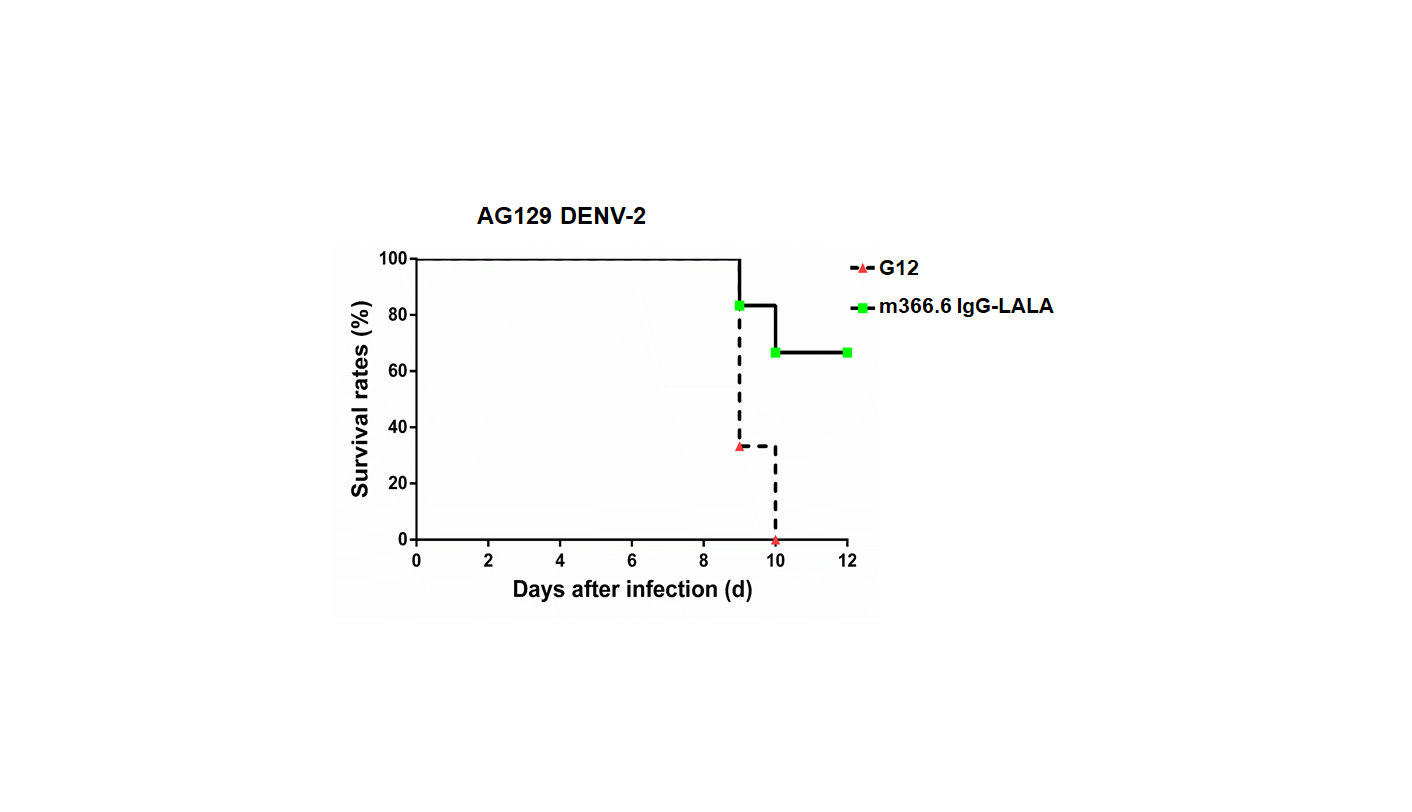

Supplement: S8 Fig — For therapeutic efficacy study, AG129 mice were treated intraperitoneally with and m366.6 IgG-LALA 16 h after viral challenge with 2x10^6^ PFU of DENV-2, and were monitored daily for 12 days for the accumulated mortality (n = 6 per group). Unrelated antibody G12 was used for the control group. (TIF) [file ppat.1007836.s008.tif]
